# Radiotherapy and MVA-MUC1-IL-2 vaccine act synergistically for inducing specific immunity to MUC-1 tumor antigen

**DOI:** 10.1186/s40425-016-0204-3

**Published:** 2017-01-17

**Authors:** Gilda G. Hillman, Lyndsey A. Reich, Shoshana E. Rothstein, Lisa M. Abernathy, Matthew D. Fountain, Kali Hankerd, Christopher K. Yunker, Joseph T. Rakowski, Eric Quemeneur, Philippe Slos

**Affiliations:** 1 Department of Oncology, Wayne State University School of Medicine, Karmanos Cancer Institute, Hudson Webber Cancer Research Center, room 515, 4100 John R, Detroit, MI 48201 USA; 2 Radiation Oncology Division, Immunology & Microbiology, Wayne State University School of Medicine, Karmanos Cancer Institute, Detroit, MI 48201 USA; 3 Present address: Department of Microbiology and Immunology, Indiana University School of Medicine at Notre Dame, South Bend, IN 46617 USA; 4 Transgene SA, Parc d’Innovation, CS80166, 67405 Illkirch-Graffenstaden Cedex, France; 5 Present address: Oncodesign, 20, rue Jean Mazen, 21076 Dijon Cedex, France

**Keywords:** MVA vector, MUC1, IL-2, Radiation, Renal Cell Carcinoma

## Abstract

**Background:**

We previously demonstrated that tumor irradiation potentiates cancer vaccines using genetic modification of tumor cells in murine tumor models. To investigate whether tumor irradiation augments the immune response to MUC1 tumor antigen, we have tested the efficacy of tumor irradiation combined with an MVA-MUC1-IL2 cancer vaccine (Transgene TG4010) for murine renal adenocarcinoma (Renca) cells transfected with MUC1.

**Methods:**

Established subcutaneous Renca-MUC1 tumors were treated with 8 Gy radiation on day 11 and peritumoral injections of MVA-MUC1-IL2 vector on day 12 and 17, or using a reverse sequence of vaccine followed by radiation. Growth delays were monitored by tumor measurements and histological responses were evaluated by immunohistochemistry. Specific immunity was assessed by challenge with Renca-MUC1 cells. Generation of tumor-specific T cells was detected by IFN-γ production from splenocytes stimulated in vitro with tumor lysates using ELISPOT assays.

**Results:**

Tumor growth delays observed by tumor irradiation combined with MVA-MUC1-IL-2 vaccine were significantly more prolonged than those observed by vaccine, radiation, or radiation with MVA empty vector. The sequence of cancer vaccine followed by radiation two days later resulted in 55–58% complete responders and 60% mouse long-term survival. This sequence was more effective than that of radiation followed by vaccine leading to 24–30% complete responders and 30% mouse survival. Responding mice were immune to challenge with Renca-MUC1 cells, indicating the induction of specific tumor immunity. Histology studies of regressing tumors at 1 week after therapy, revealed extensive tumor destruction and a heavy infiltration of CD45^+^ leukocytes including F4/80^+^ macrophages, CD8^+^ cytotoxic T cells and CD4^+^ helper T cells. The generation of tumor-specific T cells by combined therapy was confirmed by IFN-γ secretion in tumor-stimulated splenocytes. An abscopal effect was measured by rejection of an untreated tumor on the contralateral flank to the tumor treated with radiation and vaccine.

**Conclusions:**

These findings suggest that cancer vaccine given prior to local tumor irradiation augments an immune response targeted at tumor antigens that results in specific anti-tumor immunity. These findings support further exploration of the combination of radiotherapy with cancer vaccines for the treatment of cancer.

**Electronic supplementary material:**

The online version of this article (doi:10.1186/s40425-016-0204-3) contains supplementary material, which is available to authorized users.

## Background

Radiotherapy is a conventional modality for solid cancers that can achieve considerable tumor debulking, however, tumors recur locally due to radioresistance, resulting in cancer progression. Several clinical trials based on immunotherapy approaches to induce an anti-tumor immune response were not effective for advanced metastatic and bulky disease and were compromised by tumor-induced immunosuppression. A strategy which is currently under extensive investigation is to combine immunotherapy/cancer vaccines with radiotherapy to enhance both a local and systemic anti-tumor immune response [1–5]. The rationale is to reduce large tumor burdens localized in the primary tumor by radiation and eradicate local residual tumor and metastases by inducing anti-tumor immune responses with cytokine gene therapy, cancer vaccines, or immune checkpoint inhibitors [1, 2].

We previously demonstrated that tumor irradiation potentiates cancer vaccines based on *in situ* genetic modification of tumor cells in Renca renal adenocarcinoma and RM-9 prostate carcinoma syngeneic murine tumor models [6–9]. Renca tumor irradiation combined with intratumoral IL-2 cytokine adenovector gene therapy caused increased tumor destruction and infiltration of immune cells resulting in complete responses in 40–90% of the mice [6]. This combined therapy was more effective than radiation or gene therapy alone and induced specific cytotoxic T cell activity and specific tumor immunity [6]. In other studies, we also showed that tumor irradiation enhanced gene therapy using plasmids to convert tumor cells into a cancer vaccine [7–9]. Irradiation of the tumor nodule on the day preceding initiation of gene therapy showed 50% of mice with complete regression and induction of tumor-specific immunity [7]. Both CD4^+^ helper T cells and CD8^+^ cytotoxic T cells were essential for induction of an anti-tumor immune response as demonstrated by in vivo depletion of these subsets [9].

Recently, mechanistic studies on the role of radiation to enhance immunotherapy gave further insights into immune modulation of the tumor microenvironment (TME) by radiation including inflammatory responses, destruction of tumor cells, disruption of stroma and vasculature [1, 2, 10–12]. Radiation-induced changes in TME elicit *in situ* vaccination by causing immunogenic cell death through release of factors from dying tumor cells including HMGB1 [13], ATP [14], calreticulin [15], complement [16], and tumor associated antigen (TAA), which activate TAA presentation by dendritic cells (DC) and priming of tumor specific CD8^+^ cytotoxic T lymphocytes (CTLs) [1, 2, 10, 17–19]. Radiation also causes local inflammation and release of cytokines, including IL-1, TNF-α, IFN-β, IFN-γ, and chemokines, which facilitate activation of the anti-tumor immune response [18, 20, 21]. However, radiation could also cause immunosuppression by increasing regulatory T cells, PD-L1 and tumor associated M2 macrophages, which secrete IL-10 and TGF-β [22–26]. These suppressive effects could be responsible for the lack of specific and lasting anti-tumor immune response when radiotherapy is administered alone. To target immune suppression and enhance immune responses against the tumor, the immunomodulatory effects of radiation could be exploited by giving radiotherapy in conjunction with cancer vaccines.

Our previous studies of immunotherapy and radiation demonstrated an increased immune response directed against the tumor cells, but the targeted TAA were unknown [6–9]. We have now investigated whether tumor irradiation augments a specific immune response to MUC1 TAA antigen [27]. MUC1 is a large mucin glycoprotein normally expressed at the luminal surface of glandular epithelia and functions to lubricate and protect epithelial cells. In carcinomas, MUC1 is over-expressed, with aberrant glycosylation profile and localization. Its overexpression in metastatic renal cell carcinoma (RCC) and prostate carcinoma is associated with poor prognosis, therefore MUC1 has been used in different types of cancer vaccines [27–30]. It is noteworthy that MUC1 was ranked second out of 75 in a priority ranking of cancer antigens from the National Cancer Institute [31]. TG4010 is a cancer vaccine construct consisting of a recombinant, highly attenuated modified vaccinia Ankara (MVA) virus strain expressing both the human MUC1 and IL-2 genes (MVA-MUC1-IL-2) [27, 32, 33]. Initial clinical Phase I and II trials using TG4010 administered subcutaneously to prostate cancer patients showed its safety and induction of specific CD8^+^ T cells, and even stabilization of the disease in some patients [34–36]. Therapy was well tolerated with mostly grade 1 and 2 adverse events including local injection site reactions, fatigue and flu-like syndrome [36]. In metastatic RCC, treatment with TG4010 followed by IL-2 and IFN- α2a cytokines also showed stable disease in 30% of the patients and specific immune responses measured in patient’s CD4^+^ and CD8^+^ T cells [37]. Phase II trials of TG4010 in combination with chemotherapy for advanced stages of non-small cell lung cancer (NSCLC) demonstrated promising response rates and survival data [38–40]. Although this vaccine could induce immune responses and stabilization of disease in some of the patients, additional approaches to enhance its efficacy are needed.

To enhance the therapeutic effect of TG4010, i.e., MVA-MUC1-IL-2 cancer vaccine, we have tested the efficacy of tumor irradiation for the treatment of Renca murine RCC cells transfected with MUC1. We showed that only the combination of MVA-MUC1-IL-2 and tumor irradiation resulted in complete responders and induction of tumor specific immunity. This was not achieved by treatment with either modality alone. We demonstrated that administration of vaccine prior to radiation was a more effective sequence than the reverse sequence.

## Methods

### Tumor model

Renca is a murine RCC line of spontaneous origin in a BALB/c mouse which is maintained in vivo by serial intraperitoneal (i.p.) or subcutaneous (s.c.) passages [6]. Renca cells were transfected with a plasmid coding for human MUC1 and a stable Renca-MUC1 cell line was generated. Expression of MUC1 was tested by immunofluorescence using anti-human MUC1 antibody (clone S.854.6, Thermo Fisher) followed by labeling with goat anti-mouse IgG conjugated to Alexa Fluor 488. Cells were analyzed by flow cytometry [41] and showed 96.5% MUC1 positive cells in the Renca-MUC1 line, compared to 1.84% positive cells in the original Renca cells (data not shown). Renca-MUC1 cells were cultured in vitro in DMEM medium in the presence of hygromycin B for selection of stably transfected cells. For in vivo implantation, Renca-MUC1 cells were washed in PBS and injected s.c. in the right flank at 3x10^5^ cells in 0.05 ml PBS, in 4–6 week old female BALB/c mice (Harlan Sprague Dawley Inc, Indianapolis, IN). Mice were housed and handled in facilities accredited by the American Association for the Accreditation of Laboratory Animal Care. The animal protocol was approved by Wayne State University Animal Investigation Committee.

### Vaccine production

TG4010 (Transgene, SA) is a suspension of MVA–MUC1–IL-2 vector particles consisting of a recombinant, attenuated, Modified Vaccinia Ankara (MVA) virus containing the coding sequence for human MUC1 and IL-2. The MVA–MUC1–IL-2 vector (MVATG9931) was generated by homologous recombination in a subclone of MVA named N33 using transfer plasmid pTG9931, which carried the genes for MUC1 and IL-2 and flanking sequences surrounding Deletion II of MVA. MVA–MUC1–IL-2 was produced on primary chicken embryo fibroblasts. As an internal negative control, an empty MVA vector (MVATGN33.1) was used. The vectors were diluted in S08 buffer consisting of 10 mM Tris/HCl, 5% sucrose (w/v), 10 mM sodium glutamate, 50 mM NaCl (pH 8).

### Radiation

Photon irradiation was delivered at a single dose of 8 Gy to s.c. tumors located on the right flank. Three anesthetized mice, in jigs, were positioned under a 6.4 mm lead shield with 3 cut-outs in an aluminum frame mounted on the X-ray machine to permit selective irradiation of the right flank in 3 mice at a time [41]. The dose rate was 58 cGy/min and first HVL is 2.68 mm Cu. Photon irradiation was performed with a PANTAC Bipolar Series 2 HF 300 (Test Equipment Distributors, LLC) operated at 250 kV, 10 mA with 0.5 mm tin + 0.25 mm copper + 1 mm aluminum filter at a 42.0 cm target to mouse distance.

### Tumor treatment with vaccine and/or radiation

**Fig. 1:**
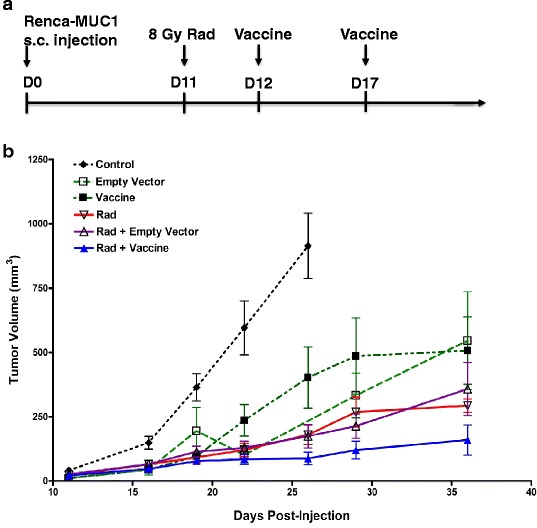
Growth curves of Renca-MUC1 tumors treated with tumor irradiation and vaccine. Established Renca-MUC1 tumors were untreated (Control) or treated with MVA-empty (Empty Vector), MVA-MUC1-IL-2 vector (Vaccine), with or without radiation (Rad). Radiation was administered at 8 Gy photons on day 11. Two peritumoral injections of vectors were administered on days 11 and 17, at 10^7^ PFU in 25 μl S08 buffer. **a**. *Schedule of treatment*. **b**. *Tumor growth curves*. Each symbol represents the mean tumor volume of 7-9 mice per group ± SEM at different time points post cell injection. Data were compiled from two experiments

Mice were treated with radiation and vaccine when Renca-MUC1 tumors reached a size between 0.3x0.4 cm and 0.3x0.5 cm, i.e., a volume of 13–15 mm^3^. Established tumors were irradiated at 8 Gy photons on day 11 (Fig. 1a). A day later, peritumoral injections of vaccine MVA–MUC1–IL-2 or MVA empty vectors were initiated using a concentration of 10^7^ PFU in 25 μl S08 buffer. A second injection of vaccine was given 5 days later, i.e., on day 17 (Fig. 1a). Experimental groups consisted of 7–9 mice/group. Mice were monitored for tumor growth and survival. Tumors were measured with a caliper in three dimensions, 3 times a week. Tumor volume was calculated using the formula: 0.5236 x length x width x height. When tumors reached 1.5 cm in greatest diameter or 1 cm with ulceration, mice were sacrificed in accordance with animal facilities regulations.

### Histology and immunohistochemistry

Tumors were resected, fixed in 10% buffered formalin, embedded in paraffin, sectioned and processed for staining with H&E. Tumor sections were stained by immunohistochemistry. Sections were blocked with IHC Tek Antibody Diluent, and then incubated with primary purified monoclonal antibodies (Abs) directed against CD45, CD4, CD8 and F4/80 (eBiosciences, San Diego, CA) followed by biotinylated secondary Abs (1:300) [41]. Staining was amplified with the avidin-biotin system immunoperoxidase technique. Tumors were examined on a Nikon E800 microscope.

### Evaluation of IFN-γ secreting T cells

To determine whether radiation and vaccine induce tumor specific T cells, splenocytes were stimulated with Renca-MUC1 lysates in plates coated with anti-mouse IFN-γ Ab. Tumor lysates were obtained by 3 cycles of thawing and freezing of Renca-MUC1 cells at 37^o^C and –80^o^C, and lysate was separated by centrifugation at 3,000 rpm for 5 min at 4^o^C. Splenocytes were isolated from mice treated with vaccines and/or radiation following challenge with Renca-MUC1 cells. Splenocytes, at 10^6^ cells/100 μl, were stimulated with 10 μg/100 μl tumor lysates in 96 well microplates pre-coated with anti-mouse IFN-γ Ab. Cells were incubated for 48 h at 37^o^C (5% CO_2_ incubator). Plates were, then, processed for detection of secreted IFN-γ using a mouse IFN-γ ELISPOT kit (eBiosciences, San Diego, CA) according to manufacturer’s instructions. The number of spots was counted using the ImmunoSpot Analyzer from CTL Analyzers, LLC (Shaker Heights, OH).

### Statistical analysis

Mean tumor volumes at defined times were analyzed with the GraphPad Prism Software (Version 6.07) using the Kruskall-Wallis test for all the treatments. A significant difference between all treatments (*P* < 0.05) was followed by pairwise comparisons using the Dunn’s multiple comparison test. Survival was analyzed using the Log-rank (Mantel-Cox) test with GraphPad Prism Software (Version 6.07). A significant difference between all treatments (*P* < 0.05) was followed by pairwise comparisons using the Log-rank (Mantel-Cox) test.

## Results

### Enhanced tumor growth inhibition by tumor irradiation and vaccine

In pilot experiments, titration experiments using radiation doses of 5 and 8 Gy and MVA-MUC1-IL-2 vaccine doses of 10^5^, 10^6^ and 10^7^ PFU showed that the combination of 8 Gy tumor irradiation with 10^7^ PFU MVA-MUC1-IL-2 was the most effective for the treatment of Renca-MUC1 s.c. tumors (Additional file 1: Figure S1 and Additional file 2: Figure S2). Whereas 5 Gy radiation alone or combined with vaccine did not cause tumor growth inhibition, tumor growth delays were observed at 8 Gy (Additional file 1: Figure S1). Combination of 8 Gy with vaccine doses of 10^6^ and 10^7^ PFU led to complete responders (Additional file 1: Figure S1, Additional file 2: Figure S2). Therefore, doses of 8 Gy radiation and 10^7^ PFU vaccine were selected to investigate the effect of single and combined therapies. Established Renca-MUC1 s.c. tumors were treated with radiation, MVA-MUC1-IL-2 vector (vaccine), MVA-empty vector or combined treatments of radiation with the vectors (Fig. 1). Radiation was administered at 8 Gy photons on day 11 and two peritumoral injections of vectors were administered on days 11 and 17, at 10^7^ PFU as shown in Fig. 1a. Treatment with vaccine only or empty vector slowed down tumor growth compared to control tumors treated with vehicle (Fig. 1b, *p* > 0.05; Additional file 2: Figure S2). Radiation alone or radiation combined with empty vector caused tumor growth delays for about 2 weeks, and then tumor regrowth occurred (Fig. 1b, Additional file 2: Figure S2). A greater inhibition of tumor growth was observed by combining tumor irradiation with the vaccine compared to radiation alone or the vaccine alone (Fig. 1b, *p* < 0.05; Additional file 2: Figure S2). It should be noted that distant s.c. delivery of the vaccine to the flank opposite to the tumor did not synergize with tumor irradiation in contrast to peritumoral injection.

### Sequence of vaccine and radiation for the treatment of Renca-MUC1 tumors

**Fig. 2:**
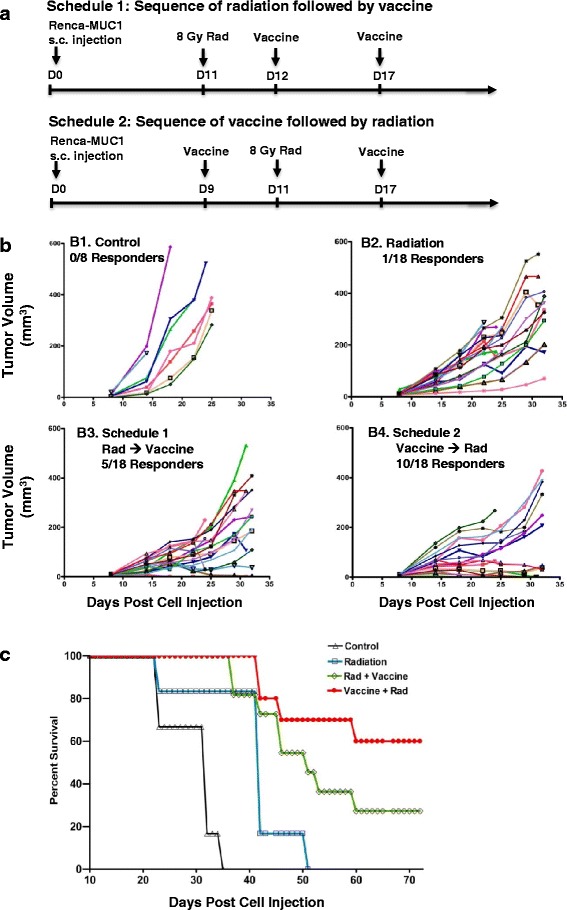
Sequence of tumor irradiation and vaccine for the treatment of Renca-MUC1 tumors. Established Renca-MUC1 tumors were treated with 8 Gy radiation (Rad) and MVA-MUC1-IL-2 vector (Vaccine) administered peritumorally at 10^7^ PFU in 25 μl S08 buffer. **a**. *Schedule 1: Sequence of radiation followed by vaccine.* Tumors were irradiated on day 11, followed by vaccine on day 12 and 17. *Schedule 2: Sequence of vaccine followed by radiation.* Tumors were first injected with vaccine on day 9, then were irradiated two days later on day 11, followed by a second treatment of vaccine on day 17. **b**. *Tumor growth curves.* **B1**. Control (Control) untreated mice. **B2**. Radiation treatment alone. **B3**. Schedule 1 sequence of radiation followed by vaccine. **B4**. Schedule 2 sequence of vaccine followed by radiation. Each symbol represents the tumor volume of individual mice with 8 mice per group for control and 18 mice per treatment group B2, B3, and B4 at different time points post cell injection. **c**. *Survival curves*. In a separate experiment identical to that shown in A, established Renca-MUC1 tumors were treated with 8 Gy radiation (Rad) and MVA-MUC1-IL-2 vector (Vaccine) administered peritumorally at 10^7^ PFU using either Schedule 1 of radiation followed by two vaccine treatments or Schedule 2 of vaccine followed by radiation and a second vaccine treatment. Mice were followed for survival (*n* = 8 mice for control or radiation alone and *n* = 12 mice for combined radiation and vaccine)

We have shown that tumor irradiation given one day prior to vaccine treatment resulted in increased inhibition of tumor growth (Fig. 1). To investigate the sequence of combined therapies and its effect on tumor response, two different sequences consisting of radiation first followed by vaccine and vaccine first followed by radiation were compared in the same experiment using 8 Gy radiation and peritumoral vaccine injections at 1 x10^7^ PFU (Fig. 2a). In the first schedule of the sequence of radiation followed by vaccine, tumors were irradiated on day 11, followed by vaccine administrations on day 12 and 17 (Fig. 2a). In the second schedule of the sequence of vaccine followed by radiation, tumors were first injected with vaccine on day 9, then were irradiated two days later on day 11, followed by a second treatment of vaccine on day 17 (Fig. 2a). Based on tumor size, the day 11 time point was selected in schedule 1 and schedule 2 as a radiation time point to keep consistency with tumor size to be irradiated (minimal differences were observed in tumor growth within the 2 day interval). Control tumors showed a rapid growth (Fig. 2B1). Treatment with radiation alone showed tumor growth delays but most of the tumors started growing again by 1–2 weeks after radiation with only 1 mouse out of 18 (5% responders) showing a continued tumor response (Fig. 2B2, *p* > 0.05). The sequence of radiation followed by vaccine caused tumor growth delays in all mice up to day 25, then varied tumor growth kinetics were observed with a marked tumor inhibition in 5 mice out of 18 (27% responders) (Fig. 2B3, *p* < 0.05 compared to control or radiation). The sequence of vaccine followed by radiation also showed significant tumor growth delays (*p* < 0.05 compared to control or radiation), and the tumor regressed in 10 mice out of 18 (55% responders) (Fig. 2B4).

In a separate and identical experiment aimed at following mouse long-term survival, established Renca-MUC1 tumors were treated with 8 Gy radiation and MVA-MUC1-IL-2 vector using either the schedule of radiation followed by two vaccine treatments (Fig. 2a, Schedule 1) or the schedule of vaccine followed by radiation and a second vaccine treatment (Fig. 2a, Schedule 2). Follow-up of mouse survival showed that all mice in the control group were dead by day 35 (Fig. 2c). Radiation alone caused an increase in median survival to 42 days compared to 32 days in control mice (Fig. 2c, *p* < 0.01). The sequence of radiation followed by vaccine caused a further increase in median survival to 52 days compared to radiation or control mice (*p* < 0.01), resulting in 30% overall survival by day 70 (Fig. 2c). The reverse sequence of vaccine followed by radiation was more effective with 60% mouse survival by day 70 (Fig. 2c, *p* < 0.01). The mouse survival corroborates the findings of tumor growth depicted in Fig. 2b. These data indicate that the sequence of vaccine followed by radiation elicits a more effective anti-tumor response compared to the sequence of radiation followed by vaccine.

### Histology and immune cell infiltration in Renca-MUC1 tumors treated with radiation and vaccine

**Fig. 3:**
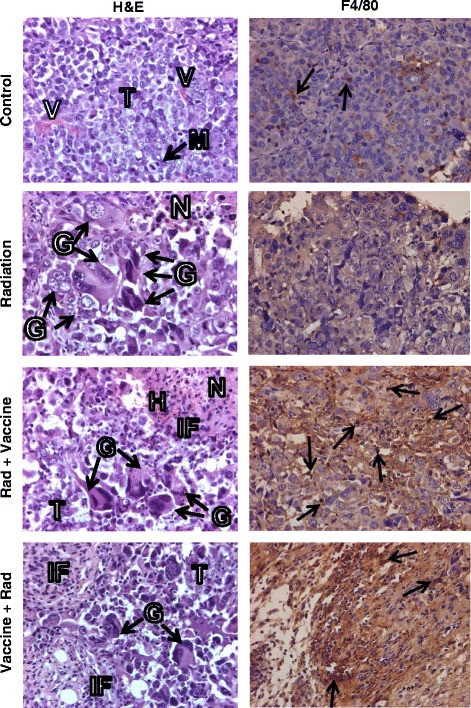
Histology and macrophage infiltration in Renca-MUC1 tumors treated with radiation and vaccine. Established Renca-MUC1 tumors were treated with 8 Gy radiation (Rad) and MVA-MUC1-IL-2 vector (Vaccine) administered peritumorally at 10^7^ PFU using either Schedule 1 of radiation followed by two vaccine treatments (Rad + Vaccine) or Schedule 2 of vaccine followed by radiation and a second vaccine treatment (Vaccine + Rad) as described in Fig. 2. Tumors sections, obtained at one week after the last vaccine treatment, were stained for H&E or by IHC for F4/80^+^ macrophages. The main findings were labeled with T for tumor, V for vessels, N for necrosis, M for mitosis, G for giant cells, H for hemorrhages and IF for inflammatory cells. In H&E stained tumor sections, control tumors presented as sheets of pleomorphic epithelial cells with frequent mitosis and minimal immune cells infiltrates. Radiation-treated tumors showed areas of necrosis, numerous giant cells with abnormal mitosis, eccentric nuclei or large vacuoles. Focal infiltration of immune cells was seen. Tumors treated with radiation and vaccine showed extensive tumor destruction with large areas of necrosis and hemorrhages, a few remaining giant tumor cells and overall decreased cellularity. Staining for F4/80^+^ macrophages showed few macrophages within control and radiation treated tumors but a heavy infiltration of macrophages in radiation and vaccine treated tumors (arrows). The extent of tumor destruction and immune infiltration was greater with Vaccine + Rad sequence than with Rad + vaccine sequence. All magnifications X40

**Fig. 4:**
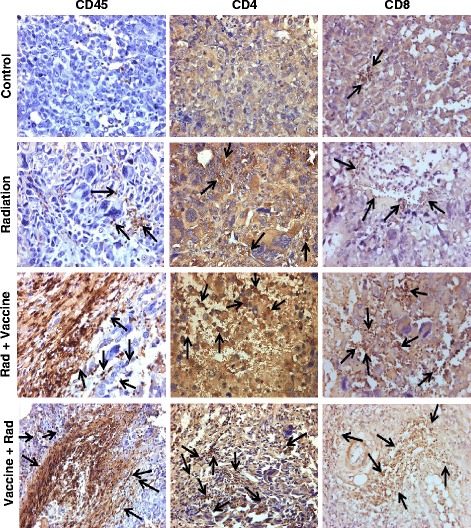
Leukocyte infiltration in Renca-MUC1 tumors treated with radiation and vaccine. Tumor sections obtained from the experiment described in Fig. 3 were also stained by IHC for CD45^+^ leukocytes, CD4^+^ T_H_ cells, and CD8^+^ CTL and cells positive for these markers are shown (arrows). Control tumors showed minimal immune cell infiltration. Radiation showed focal infiltration of CD45^+^, CD4^+^, and CD8^+^ cells. Following radiation and vaccine, a massive infiltration of CD45^+^, CD4^+^, and CD8^+^ cells was observed in areas of tumor destruction. The extent of tumor destruction and immune infiltration was greater with Vaccine + Rad sequence than with Rad + vaccine sequence. All magnifications X40

Tumors obtained at day 24, 1 week after the last vaccine treatment from the experiment described in Fig. 2, were processed for histological H&E staining or immunohistochemistry for immune cells. Untreated Renca tumors presented as large vascularized nodules consisting of sheets of pleomorphic epithelial cells with large nuclei, prominent nucleoli and frequent mitosis (Fig. 3, H&E). These tumors showed isolated F4/80^+^ macrophages and minimal infiltration by CD45^+^ leukocytes, CD4^+^ T_H_ cells, and CD8^+^ CTL (Fig. 3 and 4, Table 1). Tumors treated with vaccine alone exhibited focal areas of tumor destruction with leukocyte infiltrate (data not shown). Radiation-treated tumors showed areas of tumor necrosis with tumor debris and remaining tumor areas with multiple abnormal degenerating large giant cells containing large vacuoles and several nuclei (Fig. 3, H&E). Eccentric and pyknotic nuclei were seen in giant cells, which are characteristic of the effect of radiation on tumor cells, causing defective mitosis and cytokinesis, as previously described [6]. Focal infiltration of immune cells was seen in these tumors (Fig. 4, Table 1). In contrast, tumors treated with combined radiation and vaccine showed extensive tumor destruction with large areas of necrosis and hemorrhages (Fig. 3, H&E). A few remaining giant tumor cells were observed but a marked overall decrease in cellularity was prominent (Fig. 3, H&E). These tumors were heavily infiltrated by F4/80^+^ macrophages (Fig. 3, Table 1) and CD45^+^ leukocytes consisting of T cells positive for CD4 T_H_ and CD8 CTLs markers (Fig. 4, Table 1). The extent of tumor destruction and immune infiltration was greater with the sequence of vaccine followed by radiation than radiation followed by vaccine (Figs. 3 and 4).

**Table 1:** Histological scoring of immune cell infiltrates in tumors treated with radiation and vaccine

	Control	Radiation	Rad + Vaccine	Vaccine + Rad
F4/80^+^ cells	+	+	+++	++++
CD45^+^ cells	+	++	++++	++++
CD4^+^T_H_ cells	±	++	+++	++++
CD8^+^ CTL cells	+	++	++++	++++

Tumors were either treated with the sequence of radiation followed by vaccine (Rad + Vaccine) or vaccine followed by radiation (Vaccine + Rad) as shown in Fig. 2a. The extent of inflammatory infiltration in treated tumors, which is presented in Figs. 3 and 4, was scaled from mild (±), moderate (+), strong (+ +), very strong (+ + +), to heavy (+ + + +) for immune cell markers including F4/80+ macrophages, CD45+ leukocytes, CD4+ TH cells and CD8+ CTL

### Anti-tumor response and specific immunity to tumor rechallenge

The responses of mice treated with vaccine and radiation obtained from 3 independent experiments are summarized in Table 2. No responders were observed in control mice and in mice treated with vaccine only. In radiation-treated mice, only 4% of mice showed complete regression. Six radiation-treated surviving mice, including two cured mice and 4 mice still bearing tumors, developed tumors in their left flank upon challenge with Renca-MUC1 cells, showing that radiation alone did not cause anti-tumor immunity (Table 2). When mice were treated with radiation and empty MVA, a few mice (3 out of 20 mice) were cured and rejected Renca-MUC1 tumor challenge indicating some tumor immunity, probably directed against vaccinia antigens (Table 2). Mice treated with the sequence of radiation followed by vaccine (Schedule 1, Fig. 2a) showed a higher cure rate (24%) and demonstrated specific tumor immunity when rechallenged with Renca-MUC1 (Table 2). A few responding mice rechallenged in a different site also with Renca cells did reject Renca, suggesting induction of immunity to Renca TAA. In repeated experiments, treatment with the schedule of vaccine followed by radiation (Schedule 2, Fig. 2a) consistently resulted in a greater number of responders (58%) which were immune to rechallenge with Renca-MUC1 (Table 2).

**Table 2:** Response of Renca-MUC1 tumors to radiation and vaccine and tumor rechallenge

	^a^Responders/Cured		^b^Immune to Rechallenge	^c^IFN-γ Mean Spots ± SD	
	# Mice/Total	Percent			
Control	0/36	0%	N/A	N/A	
Vaccine	0/7	0%	N/A	N/A	
Radiation	2/43	4%	0/6	N/A	
Rad + Empty Vector	3/20	15%	3/3	140.4 ± 29.4	
Rad + Vaccine (Schedule 1)	11/45	24%	9/9	156.4 ± 24.6	
Vaccine + Rad (Schedule 2)	17/29	58%	7/8	152.6 ± 24.1	

Data were compiled from three independent experiments

^a^Responders/Cured: Responders were characterized by inhibition of tumor growth or complete tumor regression

^b^Immune to rechallenge: Responding and non-responding mice were challenged with 1x10*5* Renca-MUC1 cells injected in the left flank contralateral to the Renca-MUC1 primary tumor on day 40–60

^c^IFN-γ: Splenocytes were obtained from mice at 3-4 weeks after tumor rechallenge, stimulated with Renca-MUC1 cells in vitro and tested for production of IFN-γ in an ELISPOT assay

Immunity to rechallenge with Renca-MUC1 cells suggested the generation of specific T- cells against tumor antigens. Therefore, we assessed the production of IFN-γ in splenocytes isolated from mice, which were cured by vaccine and radiation treatments and rejected rechallenged tumor cells. Data were compared to splenocytes from non-responding tumor-bearing mice. Splenocytes from cured mice showed consistent production of IFN-γ cytokine upon in vitro stimulation with Renca-MUC1 cell lysates confirming that the treatment of radiation and vaccine induced T cells specific to the tumor cells (Table 2). Interestingly, responding mice also showed IFN-γ cytokine upon in vitro stimulation with Renca cell lysates, indicating the generation of T cells specific to Renca antigens in addition to MUC1 antigen. This might be an evidence of epitope spreading. In contrast, splenocytes obtained from tumor-bearing mice not responding to treatment did not produce IFN-γ.

We designed a separate pilot experiment to probe abscopal response (Table 3). Established primary Renca-MUC1 tumors on the right flank were treated with vaccine followed by radiation (schedule 2, Fig. 2a). Early rechallenge was given with Renca-MUC1 cells on the contralateral left flank, at 7 days after radiation and one day after the second vaccine treatment (Table 3). In 4 out of 8 mice, the primary Renca-MUC1 tumor regressed on the right flank and no growth of the challenged tumor on the left flank was observed upon follow up for up to 70 days, when mice were killed for IFN-γ assay (Table 3). The splenocytes from these mice produced IFN-γ cytokine upon in vitro stimulation with Renca-MUC1 cell lysates (Table 3). These data indicate that combined irradiation and vaccine induced an early systemic immune anti-tumor response, which caused an abscopal effect on a distant tumor. Control mice or radiation-treated mice developed both primary tumors in the right flank and rechallenge tumors in the left flank (Table 3).

**Table 3:** Abscopal effect

	^a^Responders/Cured		^b^Immune to Rechallenge	^c^IFN-γ Mean Spots ± SD
	# Mice/Total	Percent		
Control	0/7	0%	0/7	N/A
Radiation	0/8	0%	0/8	N/A
Vaccine + Rad (Schedule 2)	4/8	50%	4/4	147.2 ± 7.8

To test for abscopal effect, established Renca-MUC1tumors were treated either with vehicle (control), 8Gy radiation or vaccine followed by radiation (schedule 2, Fig. 2a). ^a^Responders/Cured: Responders were characterized by inhibition of tumor growth or complete tumor regression. ^b^At an early time point after treatment, 7 days after radiation and one day after the second vaccine treatment, mice were rechallenged with Renca-MUC1 cells on the contralateral left flank. ^c^On day 70, the splenocytes of mice immune to rechallenge were tested in IFN-γ ELISPOT assay

## Discussion

The MVA–MUC1–IL-2 cancer vaccine construct was successfully tested clinically in patients with MUC1-overexpressing malignancies in order to induce a specific anti-tumor immune response. Subcutaneous treatments with MVA-MUC1-IL-2 were well tolerated in patients with advanced RCC, prostate cancer, or NSCLC and were shown to induce MUC1 specific responses in some of the patients [34–39]. Nevertheless, clinical responses were limited when MVA-MUC1-IL-2 was given alone as monotherapy or combined with cytokines or chemotherapy, emphasizing the need to develop additional strategies to increase its efficacy.

Our preclinical studies demonstrate that radiation enhanced the efficacy of MVA-MUC1-IL-2 cancer vaccine in a murine RCC-MUC1 transfected tumor model. Significant tumor growth inhibition was observed using a single high radiation dose of 8Gy and two administrations of MVA-MUC1-IL-2 at a high dose of 10^7^ PFU, given a week apart. The sequence of cancer vaccine followed by radiation two days later resulted in 55–58% complete responders in short term experiments and 60% mouse survival in a long-term experiment, showing consistency in the response rate. This sequence was more effective than the sequence of radiation followed a day later by cancer vaccine leading to 24–30% complete responders and 30% mouse survival. Responding mice which showed complete tumor regression were immune to challenge with Renca-MUC1, indicating that these mice developed specific tumor immunity. These findings are similar to other studies demonstrating 40% complete anti-tumor responses by 8Gy tumor irradiation given in conjunction with a complex vaccine encoding for CEA as an antigen, for co-stimulatory molecules and GM-CSF in MC38-CEA transfected colon adenocarcinoma in CEA transgenic C57BL/6 mice [42]. These responses were not observed with radiation or vaccine alone akin to our data. A few mice treated which radiation and empty vector developed tumor immunity, which was probably a response to vaccinia antigens.

Extensive tumor destruction by radiation and MVA-MUC1-IL-2 vaccine was confirmed early by histological evaluation of regressing tumors at one week after treatment, showing that the synergistic effect of radiation and vaccine occurs rapidly. A massive invasion of F4/80^+^ macrophages, CD45 leukocytes, CD8^+^ CTLs and CD4^+^ T_H_ cells was observed in treated tumors, in contrast to focal staining of immune cells in radiation-treated tumors. These immuno-histology studies showed a drastic effect of the combined therapy on immune infiltration in the TME and corroborated with tumor regression and development of specific tumor immunity.

In support of these findings, the generation of specific T cells to MUC-1 antigen by combined therapy was confirmed by specific IFN-γ secretion in the splenocytes from cured mice stimulated in vitro with Renca-MUC1 lysates. Stimulation with parental Renca cell lysates in this assay also induced IFN-γ, suggesting a broader response against other TAA on Renca due to epitope spreading. This is in agreement with rejection of Renca cell challenge observed in cured mice in addition to rejection of Renca-MUC1 cells. This phenomenon of antigen cascade or epitope spreading was clearly demonstrated in other studies, which reported immune responses against epitopes distinct from the inducing antigen and were caused by radiation and cancer vaccines [42]. The complete anti-tumor responses observed in cured mice were associated with specific anti-tumor immunity. These data confirm that local tumor irradiation stimulated immune events potentiated by the cancer vaccine. These findings are in agreement with our previous studies demonstrating increased efficacy when tumor irradiation was combined with immunotherapy either with systemic IL-2 [43, 44] or intratumoral Ad-IL-2 in Renca tumors [6] or with plasmids to increase immunogenicity of RM-9 tumors [7, 9]. These responses were associated with generation of T cells specific to the tumor and induction of specific anti-tumor immunity, which required both CD4^+^ T_H_ cells and CD8^+^ CTLs [6, 9, 43]. Mechanistic studies have shown that local tumor irradiation activated CD8^+^ T cells in splenocytes, tumor-draining lymph nodes and TME [19, 45, 46]. However, radiation or immunotherapy alone were not sufficient to induce a curative anti-tumor response, probably as a result of immunosuppression induced in TME either by the tumor or the radiation [3, 45].

The abscopal effect of radiotherapy causing regression at distant metastatic sites was observed in some rare clinical cases, presumably as the result of an anti-tumor immune response. Recently, several pre-clinical and clinical studies showed that addition of immunotherapy increased abscopal responses by inducing systemic anti-tumor immunity [3, 47–49]. Abscopal effects were observed by strategies to improve cross-priming of anti-tumor T cells including stimulation of DC by Flt3-Ligand [47], treatment with GM-CSF [49, 50], DC [51], anti-PD1 [48] and anti-CTLA-4 antibodies [43, 52, 53]. In our earlier studies, the combination of systemic IL-2 with left lung irradiation at 8 Gy in a mouse Renca lung tumor model caused a significant abscopal response in the non-irradiated right lung [43]. In the current study, we also demonstrated evidence of the abscopal effect of tumor irradiation upon combination MVA- MUC1-IL-2 vaccine.

Even though, the anti-tumor immune response mediated by the combined therapy was significant in 50–60% of the mice, tumor regrowth in non-responders could be due to their inability to overcome immune suppression in TME. This therapeutic approach could be further improved by approaches to reduce immunosuppressive mechanisms in the TME, such as targeting immune checkpoints. Monoclonal antibodies directed against immune checkpoints including CTLA-4, PD1 or PD-L1 were found to be effective at potentiating radiotherapy, leading to enhanced primary responses and abscopal systemic responses in tumor models in mice [25, 48, 52, 54]. Interestingly, these studies showed a decrease in CD4^+^CD25^+^FOXP3^+^ immunosuppressive Tregs which was dependent on the radiation doses.

## Conclusions

Several critical issues have to be addressed when designing clinical protocols of radiation and immunotherapy including the selection of the radiation regimen and dose, the sequence and timing of both modalities, the frequency of administration and route of delivery of immunotherapy. The selection of the radiation regimen has to be considered such as fractionated radiation at low doses or fewer high radiation dose, as currently used in hypofractionation [2, 55]. In our pre-clinical studies and others, tumor irradiation with 8 Gy or doses in the range of 6–15 Gy was synergistic with immunotherapy approaches. Intratumoral versus systemic administration of immunotherapy has to be carefully selected depending on the approach. While systemic injections of cytokines and antibody therapy were found to enhance radiotherapy, some cancer vaccines were more effective when delivered in the tumor. The sequence of radiation and immunotherapy may also vary depending on the immunotherapy approach and the timing is crucial to take advantage of immune events triggered by each modality without compromising an ongoing immune response by adding either one at the wrong time, as discussed by Kalbasi et al. [55] and Vatner et al. [2]. In the current study, the anti-tumor response was more effective when the cancer vaccine preceded tumor irradiation by two days, probably by initiating immune mechanisms potentiated by subsequent immunogenic cell death caused by radiation. It should be noted that human MUC1 is a self-molecule in human beings but an immunogenic xeno-antigen in mice. Immune responses to a self- antigen are different from those to a foreign antigen; therefore our findings in the MUC1 mouse model cannot predict whether MVA- MUC1-IL-2 vaccine is a good candidate for therapeutic combination with radiation in cancer patients. Translation from mouse studies to human studies has to take into accounts the limitations of the pre-clinical experimental models and can be used only as guidance for the design of clinical protocols. Nevertheless, further pre-clinical studies investigating strategies to improve the combination of radiation with immunotherapy are warranted while conducting side by side clinical trials to address critical issues relevant for effective application of these two modalities [2, 55].

## Additional files

Additional file 1: Figure S1. Pilot experiments to determine radiation and vaccine doses for combination treatment. Established Renca-MUC1 tumors were untreated (Control) or treated with radiation at 5 or 8 Gy, or with radiation and MVA-MUC1-IL-2 vector (Vaccine) at 10^5^ or 10^6^. Radiation was administered on day 11 and two peritumoral injections of vectors were administered on days 11 and 17. Each symbol represents the tumor volume of individual mice for each treatment group of (*n* = 5–7 mice for A, B, C; *n* = 8-9 mice for D, E, F) at different time points post cell injection. Data were compiled from two experiments. (PDF 452 kb)
Additional file 2: Figure S2. Growth curves of Renca-MUC1 tumors treated with tumor irradiation and vaccine. Detailed tumor growth representing the tumor volume of individual mice for each treatment group shown in Fig. 1. (PDF 458 kb)

## Abbreviations

Abs: Antibodies
Ad-IL-2: Adenoviral vector expressing IL-2 gene
CD4^+^ cells: Helper T cells
CD45^+^ cells: Leukocytes
CD8^+^ cells: Cytotoxic T cells
CEA: Carcinoembryonic antigen
CTLA-4: cytotoxic T-lymphocyte-associated protein 4
DC: Dendritic cells
DMEM: Dulbecco's modified eagle medium
F4/80^+^ cells: Macrophages
Flt3-ligand: Fms-related tyrosine kinase 3 ligand
GM-CSF: Granulocyte macrophage colony stimulating factor
Gy: Gray
H&E: Hematoxylin eosin
HMBG1: High mobility group box 1
IFN-α2a: Interferon alpha 2a
IFN-β: Interferon beta
IFN-γ: Interferon gamma
IHC: Immunohistochemistry
IL-1: Interleukin 1
IL-10: Interleukin 10
IL-2: Interleukin 2
MUC1: Mucin glycoprotein antigen
MVA: Modified vaccinia Ankara virus
MVA-MUC1-IL2: Cancer vaccine (Transgene TG4010) modified vaccinia Ankara virus expressing human MUC-1 and IL-2 genes
NSCLC: Non-small cell lung cancer
PBS: Phosphate buffered solution
PD1: Programmed death 1
PDL1: Programmed death ligand 1
PFU: Plaque forming unit
RCC: Renal cell carcinoma
Renca: Murine renal adenocarcinoma tumor cells
Renca-MUC1: Renca cells transfected with MUC1
RM-9: Murine prostate tumor model
TAA: Tumor associated antigens
TGF-β: Transforming growth factor beta
TME: Tumor microenvironment
TNF-α: Tumor necrosis factor alpha
