# Supplementary figures and images for: Radiotherapy and MVA-MUC1-IL-2 vaccine act synergistically for inducing specific immunity to MUC-1 tumor antigen
Source: J Immunother Cancer. 2017 Jan 17;5:4. doi: 10.1186/s40425-016-0204-3 (PMC5240430; doi:10.1186/s40425-016-0204-3)

## Supp Figure 1

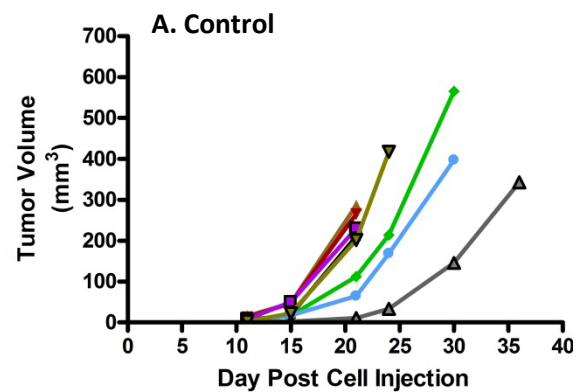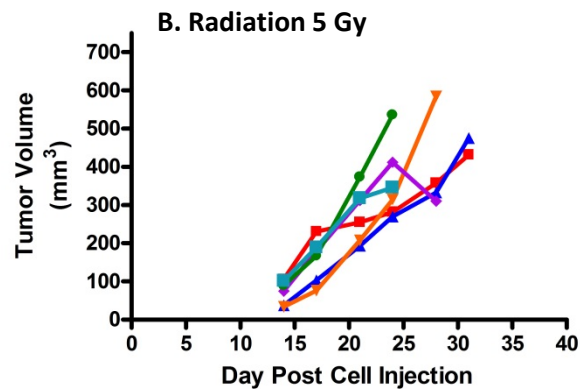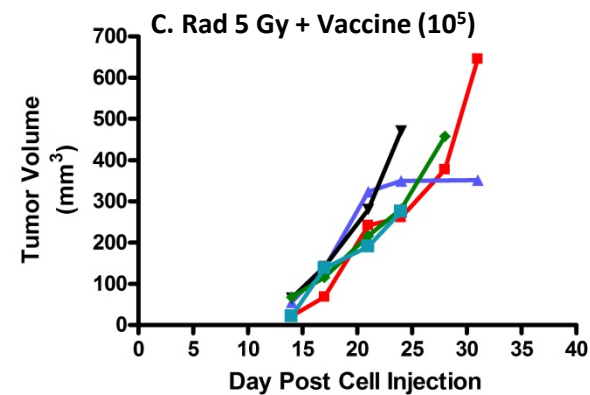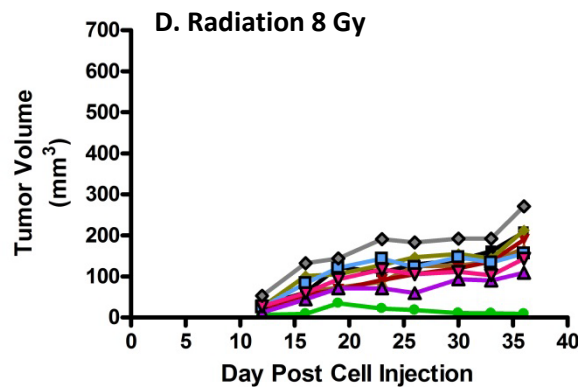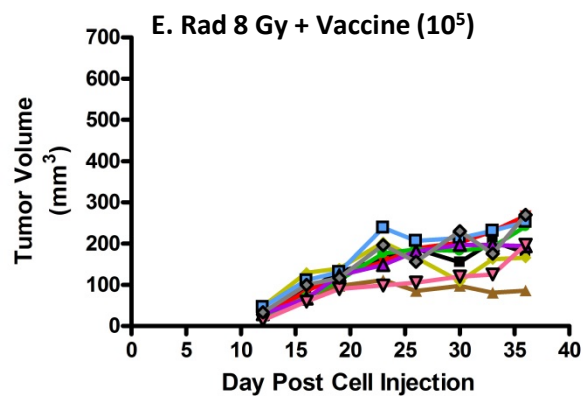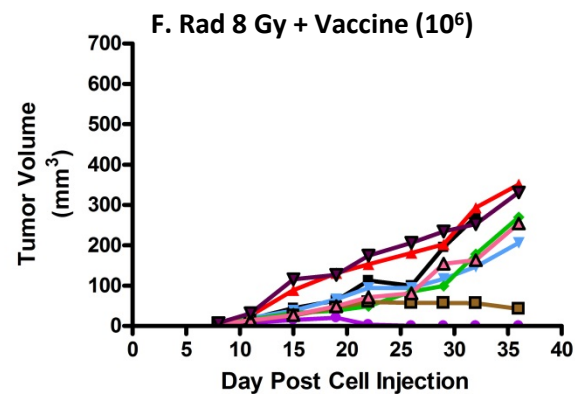

Supplement: Additional file 1: Figure S1. — Pilot experiments to determine radiation and vaccine doses for combination treatment. Established Renca-MUC1 tumors were untreated (Control) or treated with radiation at 5 or 8 Gy, or with radiation and MVA-MUC1-IL-2 vector (Vaccine) at 105 or 106. Radiation was administered on day 11 and two peritumoral injections of vectors were administered on days 11 and 17. Each symbol represents the tumor volume of individual mice for each treatment group of (n = 5–7 mice for A, B, C; n = 8-9 mice for D, E, F) at different time points post cell injection. Data were compiled from two experiments. (PDF 452 kb) [file 40425_2016_204_MOESM1_ESM.pdf]

## Supp Figure 2

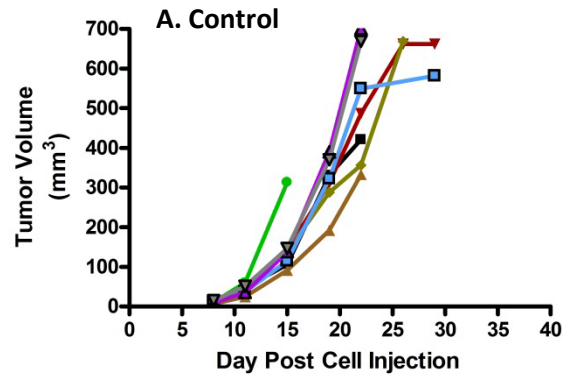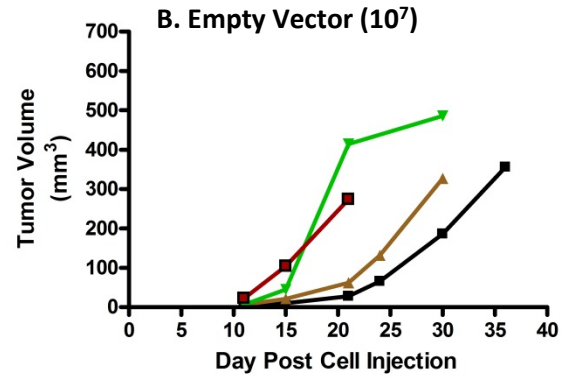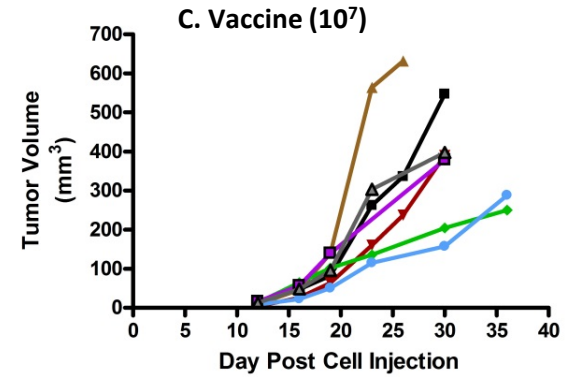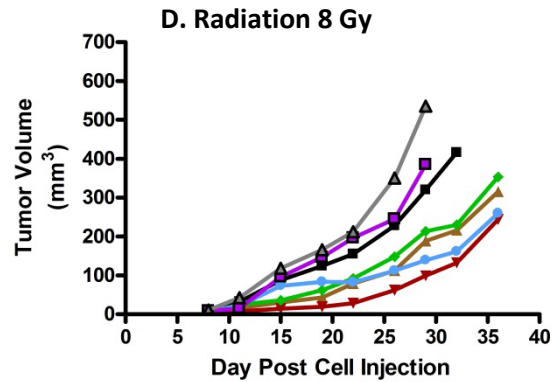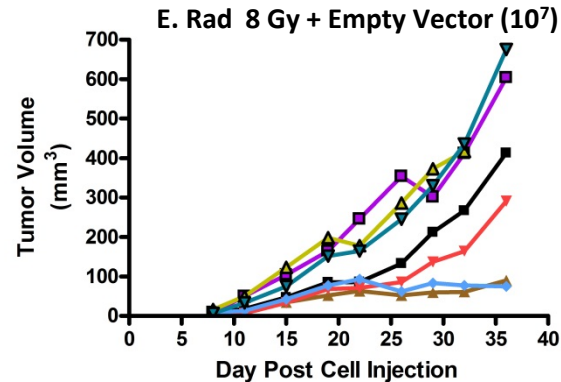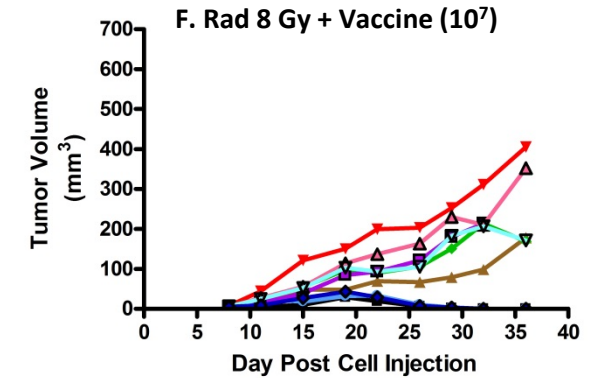

Supplement: Additional file 2: Figure S2. — Growth curves of Renca-MUC1 tumors treated with tumor irradiation and vaccine. Detailed tumor growth representing the tumor volume of individual mice for each treatment group shown in Fig. 1. (PDF 458 kb) [file 40425_2016_204_MOESM2_ESM.pdf]
